# Vitamin D Deficiency in Professional Football Players during Competitive Season of Italian First Division (Serie A)

**DOI:** 10.3390/sports12060153

**Published:** 2024-05-29

**Authors:** Marco Alfonso Perrone, Massimo Pieri, Giuseppe Caminiti, Wahid Ali, Sergio Bernardini, Attilio Parisi, Ferdinando Iellamo, Rosario Barone, Pasquale Farsetti

**Affiliations:** 1 Department of Clinical Sciences and Translational Medicine, University of Rome Tor Vergata, 00133 Rome, Italy; iellamo@uniroma2.it (F.I.); farsetti@med.uniroma2.it (P.F.); 2 Department of Experimental Medicine, University of Rome Tor Vergata, 00133 Rome, Italy; bernardini@med.uniroma2.it; 3 Department of Human Science and Promotion of Quality of Life, San Raffaele Open University, 00166 Rome, Italy; giuseppe.caminiti@uniroma5.it; 4 Department of Pathology, King George’s Medical University, Lucknow 226003, Uttar Pradesh, India; aliwahid78@gmail.com; 5 Department of Movement, Human and Health Sciences, University of Rome Foro Italico, 00135 Rome, Italy; attilio.parisi@uniroma4.it; 6 Department of Biomedicine, Neurosciences and Advanced Diagnostics, University of Palermo, 90127 Palermo, Italy; rosario.barone@unipa.it

**Keywords:** vitamin D, professional football players, exercise training, competitive season, performance, sunlight exposure

## Abstract

Background: Data in the literature have demonstrated the crucial role that vitamin D plays in the human organism, and recent studies also emphasize this essential role of vitamin D in athletes. Indeed, vitamin D acts on the skeletal muscles and plays a fundamental role in numerous physiological processes involved in immune function. Many factors such as sun exposure, skin tone, body mass index and chronic illness affect vitamin D levels. The aim of the study is to evaluate vitamin D levels in professional football players in Italy and investigate the variations in vitamin D values in footballers who train at different latitudes. Methods: The study performed is a retrospective observational study analyzing 25-OH vitamin D values in professional football players of the Italian First Division (Serie A). Two teams during the competitive season were selected: team A (latitude of 41° N in southern Italy) and team B (latitude of 45° N in northern Italy). Three time periods were identified and were classified as follows: the first quarter (May, June, July, and August), the second quarter (September, October, November, and December) and the third quarter (January, February, March, and April). The purpose of this was to study the average values of vitamin D during the year corresponding to different levels of sunlight exposure. Each athlete was subjected to at least one sampling during the three quarters of the competitive season. Results: Both vitamin D insufficiency (10.1%) and overt deficiency (1.93%) were found in Italian Serie A players. Insufficient vitamin D values are between 20 ng/mL and 29 ng/mL and overt deficiency values <20 ng/mL. At the same time, the data demonstrated a significant variation in vitamin D values depending on the period of the competitive season and the latitude of the cities of the two teams. In detail, there was no significant difference in the first quarter, while there was a significant increase in vitamin D values in team B in the second and third quarter, at *p* < 0.01 and *p* < 0.05, respectively. Conclusions: Latitude and seasons have a significant impact on vitamin D levels. Therefore, it is essential to measure vitamin D in professional football players, especially during the spring and winter months, so as to monitor changes in levels in relation to the season and latitude and evaluate any supplements. Further studies should be performed to evaluate the relationship between vitamin D deficiency and football players’ athletic performance.

## 1. Introduction

Vitamin D plays a pivotal role in bone mineral homeostasis and is involved in the regulation of a large number of physiological processes such as immune function, protein synthesis, inflammatory response and cell growth [1]. The active form of vitamin D regulates over 900 gene variants [2]. Vitamin D is obtained through skin synthesis, diet, or supplementation. About 80–90% of vitamin D is absorbed through the skin via sunlight, while the rest comes from the diet [3]. Recent studies have suggested that twenty minutes of daily sunshine with over 40% of skin exposed is required to prevent vitamin D deficiency [4]. Insufficient exposure to sunlight is a common cause of vitamin D deficiency; however, hypovitaminosis D may also be caused by other factors such as certain malabsorption syndromes or certain medications that induce hepatic p450 enzymes that accelerate vitamin D degradation [5]. Vitamin D deficiency is a major global public health issue: about 1 billion people worldwide have vitamin D deficiency, while 50% of the population has vitamin D insufficiency [6]. Interestingly, the prevalence of vitamin D deficiency is 35% higher in obese subjects irrespective of latitude and age [7]. Many factors can alter the metabolism and bioavailability of vitamin D, including variations in sun exposure due to latitude, season, time of day, atmospheric components, clothing, sunscreen use, as well as age and several chronic illnesses [8]. In particular, data from the literature suggest that people with darker skin tones and those who are overweight or have overt obesity have a high risk of vitamin D deficiency [9]. Specifically, the discovery of the vitamin D receptor (VDR) in human skeletal muscle cells [10] and the potential roles of vitamin D in muscles as regards regulating protein synthesis [11] have led to much interest in analyzing vitamin D deficiency in athletes. Some recently conducted studies have demonstrated that in athletes, vitamin D levels above the normal reference range of the general population improve skeletal muscle function, decrease recovery time during training and matches, increase net force and overall power production, and increase testosterone production [2]. However, vitamin D deficiency can also exist in healthy people, such as professional athletes [12]. In fact, screening for vitamin D in athletes may also be important, since vitamin D is involved in many immune system activation pathways, and an overt deficiency may predispose one to the development of infections among these subjects [13]. Several studies have demonstrated a high prevalence of vitamin D insufficiency in footballers, especially in the winter months, with possible implications for performance and injury outcomes [14,15].

Football players train for several hours outdoors daily, and hence are exposed to sunlight during this period; however, exposure to sunlight changes dramatically during the seasons of the year. Accordingly, some studies have focused much attention on correlating vitamin D levels and athletic performance in football players. In fact, increasing vitamin D may moderately improve aerobic performance in football players subjected to high-intensity training [16] and may ameliorate muscle strength and sprinting capacity [17]. Furthermore, there is a relationship between vitamin D and maximal oxygen uptake (VO_2_max) in professional football players [18]. However, in these athletes, the correlation between vitamin D values and sun exposure at different latitudes is still poorly understood. Therefore, this study aims to evaluate the vitamin D levels in professional football players in the Italian first division (Serie A) in relation to latitude and sun exposure.

## 2. Materials and Methods

Two Italian professional teams during a competitive season of the Italian first division (Serie A) were selected. The cities of the two teams were located at different latitudes. The players of one team train and live at a latitude of 41° N in southern Italy (team A) and the athletes of the other team train at a latitude of 45° N in northern Italy (team B). We divided the withdrawals collected into 3 periods (three quarters)—the first quarter of the competitive season (including May, June, July, and August), the second quarter of the competitive season (including September, October, November, and December) and the third quarter of the competitive season (including January, February, March, and April)—to study the average values of vitamin D during the year corresponding to sunlight exposure. This distribution took place in consideration of the Serie A competitive season and exposure to the sun in relation to the climatic seasons. Each athlete was subjected to at least one sampling during the three quarters of the competitive season. This was a retrospective observational study that analyzed 25-hydroxyvitamin D (25(OH)D) values in the plasma of Italian professional football players. We enrolled 46 males (23 in team A and 23 in team B) from two different teams of the Italian first division (Serie A) during a competitive season. In Italy, usually the competitive season starts in the last week of August and ends in May, while the pre-season (with athletic training and without official competitions) starts in July. The sampling was carried out at rest and in the morning via venous sampling. We collected and analyzed a total of 292 samples, 132 from team A and 140 from team B. In detail, from team A we collected 46 samples in the first quarter, 44 in the second quarter and 42 in the third quarter. In team B, we collected 45 samples in the first quarter, 47 samples in the second quarter, and 48 samples in the third quarter. Blood was obtained by standard venipuncture in plain tubes for serum and K2-EDTA tubes for plasma, and it was analyzed immediately after blood withdrawal. The samples were centrifuged at 2000 g for 10 min and processed immediately afterwards at the Department of Laboratory Medicine of the University Hospital Tor Vergata. Serum 25(OH)D was quantified by routine clinical laboratory methods (Vista, Centaur and Immulite Siemens Healthcare Diagnostic, Milan, Italy). The assay had a limit of quantitation of 4.2 ng/mL (10.50 nmol/L) and an assay range of 4.20–150.0 ng/mL (10.50–375.00 nmol/L). Following the manufacturer’s indications and in accordance with national guidelines, we considered normal values of vitamin D between 30 ng/mL and 100 ng/mL. Insufficient vitamin D values are between 20 ng/mL and 29 ng/mL, and overt deficiency values are <20 ng/mL [3,19,20,21,22].

Those athletes who at that time had an injury or were taking medications were excluded from each withdrawal. Vitamin D supplementation was not taken by any athlete. The athletes underwent various withdrawals during the year, varying from 8 to 10 withdrawals in the various months depending on the provisions of the medical division of the team. For this reason, we have grouped the months of the competitive season into 3 quarters according to the climatic seasons and sunlight exposure, as described before.

The athletes of the two teams had similar exercise training loads regarding the outdoor and indoor regimens. Specifically, during the week, in addition to official matches, the football players carried out 5 outdoor training sessions of approximately 2 h each time (mainly aerobic and technique training), and 2 indoor training sessions of approximately 1 h each time (mainly strength training).

The study was approved by the Ethics Committee of the University Hospital Tor Vergata (ID number 41.17), and all subjects signed informed consent. The study was conducted in accordance with the Declaration of Helsinki.

### Statistical Analysis

All data were analyzed with IBM SPSS Statistics software v.17.0 (IBM, Armonk, NY, USA). The normality of the collected data was tested using the Kolmogorov–Smirnov test. To compare values of serum concentration of 25(OH)D in the different quarters, we used the one-way ANOVA analysis and Bonferroni’s post hoc analysis. Statistical significance was set at *p* < 0.05. The graphs were made using Origin 6.1 software (OriginLab Corporation, Northampton, MA, USA).

## 3. Results

The athletes of the two teams we examined were homogeneous with regard to age and BMI. The mean values for age were 27.4 ± 4.0 and 28.7 ± 3.0 years (*p* > 0.05) for team A and team B, respectively. Regarding BMI, the mean values were 22.7 ± 0.86 and 22.2 ± 1.15 (*p* > 0.05).

**Figure 1:**
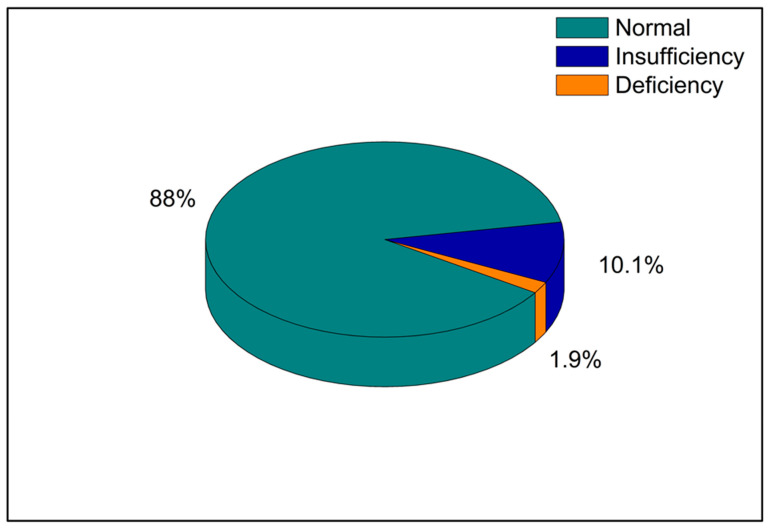
Percentages of venous samples with average normal, insufficient, and deficient vitamin D values during the entire competition season.

We first evaluated the baseline vitamin D values in all the enrolled players to analyze the general status of the serum vitamin D concentration. The baseline blood sample analysis showed that 88% had vitamin D levels within the reference range, 10.1% had vitamin D insufficiency, while 1.9% had overt vitamin D deficiency (Figure 1).

**Figure 2:**
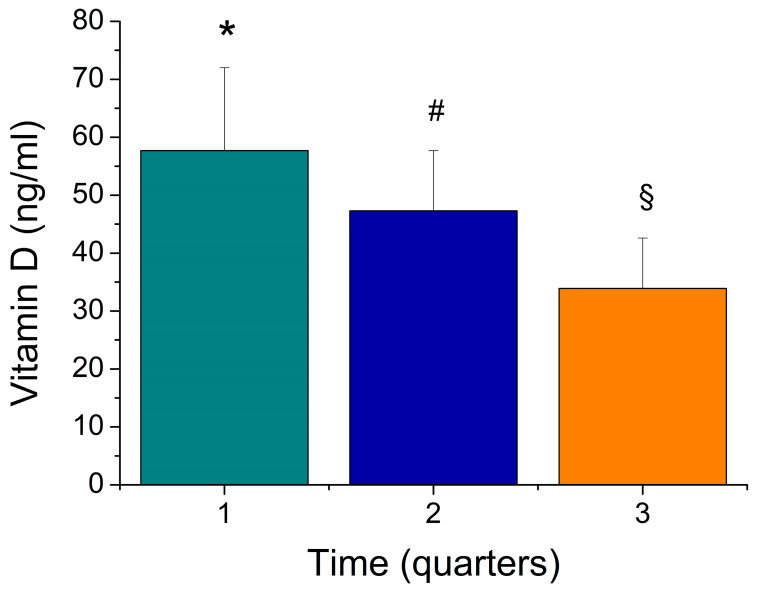
Mean values ± standard deviation of vitamin D values of first, second and third quarter of the competitive season. * *p* < 0.01 versus Time 2 and 3; # *p* < 0.01 versus Time 1 and 3; § *p* < 0.01 versus Time 2 and 3.

We then evaluated the values of vitamin D in the three quarters that were taken into consideration during the season. The statistical analysis showed that the values for the first quarter were significantly higher than in the second quarter (*p* < 0.01) and in the third quarter (*p* < 0.01). The values in the second quarter were significantly higher than in the third quarter (*p* <0.01) (Figure 2).

Our data demonstrate that there was a progressive lowering of the vitamin D levels during the competitive season in the subjects from both teams. The average values of vitamin D during the first, second and third quarters of the competitive season were equal to 50.7 ± 12.8 ng/mL, 40.4 ± 9.7 ng/mL and 34.3 ± 9.0 ng/mL, respectively (Figure 2).

**Figure 3:**
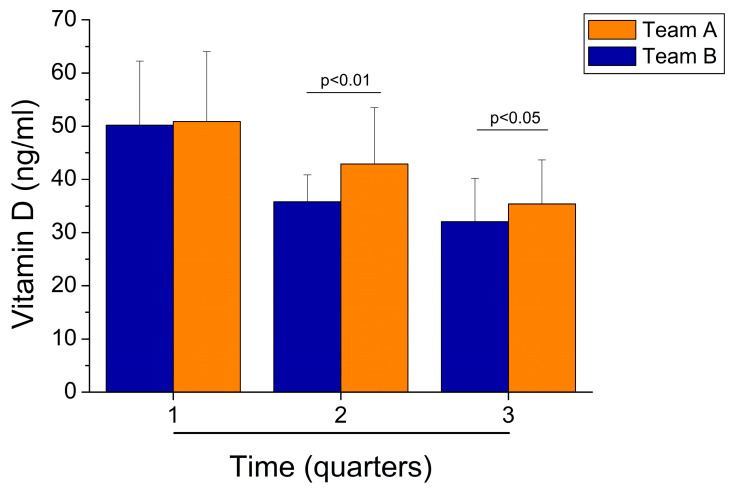
Blood values of vitamin D in team A (latitude of 41° N in southern Italy) and team B (latitude of 45° N in northern Italy) during the three quarters of the competitive season.

Thereafter, we compared the vitamin D values between the players of the two different teams in the three quarters. The data show that footballers residing at latitude 41° N in southern Italy (team A) had higher vitamin D values than athletes residing at a latitude of 45° N in northern Italy (team B) in the second and third quarters. However, there was no significant difference between the values of the two teams in the first quarter (Figure 3).

During the first four months of the competitive season, no significant difference in vitamin D values between team A and team B was demonstrated (50.9 ± 13.1 vs. 50.2 ± 12.0 ng/mL). On the other hand, during the second (42.9 ± 10.6 vs. 35.8 ± 5.0 ng/mL) and third (35.4 ± 9.3 vs. 32.0 ± 8.2 ng/mL) quarters of the competitive season, the vitamin D values were statistically higher in the team A players than in the team B players (*p* < 0.01 and *p* < 0.05, respectively) (Figure 3).

## 4. Discussion

The overall vitamin D values measured in players from both football teams were as follows: 88% of subjects were within the reference range, 10.1% had vitamin D insufficiency and the residual 1.9% had overt vitamin D deficiency, thus indicating that vitamin D levels were lower than normal in 12.0% of cases (Figure 1). The study of Lombardi et al. [23] analyzed vitamin D values over a period of 2 years in football players who belonged to three football teams of the Italian second division (Serie B) with residency between 41° and 42° N of latitude. The study demonstrated that vitamin D levels below the normal range were present in 15.7% of subjects, a percentage of insufficiency slightly higher than that reported in this study [23]. In the study conducted by Lombardi et al., the differences between the three periods were all statistically significant (*p* < 0.05), and the athletes had vitamin D values within the reference range during the first period and the second period, but low values in the third period [23]. Recent data from the literature have demonstrated that vitamin D levels should be at least 40 ng/mL for optimal physical conditions among athletes [24,25].

The athletes of the two teams we examined were homogeneous with regard to age and BMI. In addition, the athletes of the two teams can be considered superimposable, because both teams at the time of the study played in the Italian first division and the exercise training sessions included the same distribution between outdoor and indoor sessions. At each stage of the season, players spend several hours training outdoors, and therefore one would assume that latitude influence is trivial during the summer period. Rather, in the winter, autumn and spring periods, the influence of latitude has a significant impact on the synthesis of vitamin D in professional football players. Interestingly, the lowest vitamin D values in both teams were recorded in the spring, but the biggest difference between the two teams was seen during the second quarter. The reduction in vitamin D levels over time is due to climatic factors; however, recent studies have shown that football players display a higher consumption of the vitamin D metabolite compared to the general population due to the physical stress induced by intense training [26,27]. The underlying pathophysiological mechanism remains to be elucidated; however, the higher degree of vitamin D consumption is likely linked to augmented muscular activity. The reduction in vitamin D during the winter months can become problematic for footballer’s health, since vitamin D plays an important role in the function of the immune system, and athletes are prone to viral and bacterial infections following long-lasting or particularly intense workouts [28]. In fact, beyond the known functions of vitamin D in supporting bone mineralization through calcium homeostasis, promoting skeletal muscle regeneration and mitochondrial health, it has been shown that vitamin D intervenes in innate and adaptive immunity by controlling the growth and differentiation of various immune cells such as T and B lymphocytes, macrophages and dendritic cells [29]. Furthermore, studies have demonstrated that vitamin D also influences physical performance [24,25] in athletes, as well as playing an essential role in metabolism and muscle recovery. As a result, vitamin D deficiency could cause muscle fatigue and muscle injuries, with the latter being more frequent in fatigued subjects [30]. Vitamin D levels are associated with neuromuscular performance and aerobic capacity in professional football players, and there is a linear relationship between vitamin D levels, jumping performance, and speed [16,18]. Vitamin D could also influence VO_2_max through the effects on erythropoiesis, possibly affecting iron metabolism, by modulating the inflammatory response, and increasing erythropoietin resistance [16,18]. As aforementioned, it is recommended that vitamin D values be higher than 40 ng/mL, a value considered by several physiologists as the minimum threshold necessary to obtain optimal performance [24,25]. In both teams, the average values of vitamin D were lower than 40 ng/mL during the third quarter of the competitive season, and in the players of team B the average value of vitamin D was lower than 40 ng/mL in the second quarter of the competitive season. The winter and spring periods of the competitive season are among the busiest of the season, because there are both league games and national and international cups. Moreover, the third quarter of the season is very important because it is the final phase of all competitions. The vitamin D levels of the players we tested from May to August (50.9 ± 13.1 ng/mL for team A and 50.2 ± 12.0 ng/mL for team B) were much higher than the average value of vitamin D in players of three Greek professional teams (found to be equal to 34.4 ng/mL; in this study, the authors took venous samples during the summer) [18] and higher than the average value of vitamin D (41.8 ng/mL) during the month of August of players of a team in the Premier League (latitude 51° N) [31]. We also compared our relevant data during the winter (42.9 ± 10.6 ng/mL for team A and 35.8 ± 5.1 ng/mL for team B) with the aforementioned group of players of the Premier League, which showed an average value of 20.4 ng/mL [31]. These differences compared to the English and Greek data could be attributable to both different latitudes and different nutrition patterns. Other authors have not stated average values in their studies, but rather have reported the percentage of vitamin D insufficiency and overt deficiency in different periods of the year. According to a Spanish study involving 34 healthy professional football players from two First Division teams of Andalusia (Southern Spain), located at 37° N latitude, it was shown that 93% of the professional football players had sufficient vitamin D in mid-October (≥30 ng/mL) and 64% of the same players showed low levels of vitamin D in February (<30 ng/mL) [32]. We can therefore hypothesize that during the winter, the vitamin D values were higher in the players we analyzed. A Russian study involving youth players (who live and train at 55° N latitude) showed that in December, vitamin D insufficiency and deficiency was highly prevalent in the analyzed population, with a reduced vitamin D plasma concentration observed in 42.8% of participants. Vitamin D insufficiency was found in 19.9% of the participants, and vitamin D deficiency was found in 22.9% of the participants. Vitamin D level was within the normal range in 26.7% of young soccer players, while in 30.5% of the players, it reached 61–130 ng/mL, and was above reference values [24]. This very high incidence of vitamin D insufficiency and deficiency in Russian footballers compared to ours could be attributable to the large difference in latitude and possible reduced outdoor training sessions in winter due to low temperatures. The same authors prescribed daily cholecalciferol supplementation in footballers with low vitamin D values, showing a significant increase in values at 30 and 60 days [24]. Athletes should be constantly monitored for serum levels of 25(OH)D throughout the year, and supplementation with vitamin D may be an important factor that supports the training process and contributes to physical ability, especially in the period when deficits are observed [17,33,34,35]. Furthermore, an interesting study demonstrated that an appropriate level of vitamin D decreases the incidence of stress fractures in athletes [36]. The same study also demonstrated that, in the case of low vitamin D values, oral supplementation decreases the risk of stress fractures [36]. Vitamin D supplementation, sunlight radiation and monitoring vitamin D levels are important factors to consider during a football season that allow the athlete to maintain good performances [37,38]. Monitoring vitamin D can be useful for identifying potential declines, and, if necessary, providing individual supplementation plans for athletes [39].

The limitations of this study include the lack of information about the diet and skin pigmentation of footballers, factors that can influence vitamin D values, albeit minimally compared to sun exposure. Another limitation of the study is not having information about the athletic performance and injuries of footballers to correlate with vitamin D values.

## 5. Conclusions

Vitamin D insufficiency and deficiency have been found in Italian Serie A football players, especially during the autumn/winter months and early spring months. Latitude has an important impact on vitamin D levels in both autumn/winter and spring, but not in summer. Therefore, it is essential to measure vitamin D values in professional footballers, especially in those months. If vitamin D insufficiency or deficiency is found, footballers may be prescribed supplementation to allow athletes to prevent the onset of infections, reduce the risk of muscle injuries, and train and compete to the best of their ability.

## Data Availability

The data presented in this study are available on request from the corresponding author due to privacy and ethical restrictions.
